# Lifestyle factors affecting gastroesophageal reflux disease symptoms: a cross-sectional study of healthy 19864 adults using FSSG scores

**DOI:** 10.1186/1741-7015-10-45

**Published:** 2012-05-03

**Authors:** Nobutake Yamamichi, Satoshi Mochizuki, Itsuko Asada-Hirayama, Rie Mikami-Matsuda, Takeshi Shimamoto, Maki Konno-Shimizu, Yu Takahashi, Chihiro Takeuchi, Keiko Niimi, Satoshi Ono, Shinya Kodashima, Chihiro Minatsuki, Mitsuhiro Fujishiro, Toru Mitsushima, Kazuhiko Koike

**Affiliations:** 1 Department of Gastroenterology, Graduate School of Medicine, The University of Tokyo, 7-3-1, Hongo, Bunkyo-ku, Tokyo, Japan; 2 Kameda Medical Center Makuhari, CD-2, 1-3, Nakase, Mihama-ku, Chiba-city, Japan

**Keywords:** gastroesophageal reflux disease (GERD), FSSG (Frequency Scale for the Symptoms of GERD), quality of sleep, dietary habits, proton pump inhibitor (PPI), histamine H_2_-receptor antagonist (H_2_RA)

## Abstract

**Background:**

Gastroesophageal reflux disease (GERD) is a very common disorder worldwide, comprised of reflux esophagitis (RE) and non-erosive reflux disease (NERD). As more than half of GERD patients are classified into the NERD group, precise evaluation of bothersome epigastric symptoms is essential. Nevertheless, compared with many reports targeting endoscopic reflux esophagitis, large-scale studies focusing on GERD symptoms have been very scarce.

**Methods:**

To elucidate lifestyle factors affecting GERD symptoms, 19,864 healthy adults in Japan were analyzed. Sub-analyses of 371 proton pump inhibitor (PPI) users and 539 histamine H_2_-receptor antagonist (H_2_RA) users were also performed. Using the FSSG (Frequency Scale for the Symptoms of GERD) score as a response variable, 25 lifestyle-related factors were univariately evaluated by Student's *t*-test or Pearson's correlation coefficient, and were further analyzed with multiple linear regression modelling.

**Results:**

Average FSSG scores were 4.8 ± 5.2 for total subjects, 9.0 ± 7.3 for PPI users, and 8.2 ± 6.6 for H_2_RA users. Among the total population, positively correlated factors and standardized coefficients (β) for FSSG scores are inadequate sleep (β = 0.158), digestive drug users (β = 0.0972 for PPI, β = 0.0903 for H_2_RA, and β = 0.104 for others), increased body weight in adulthood (β = 0.081), dinner just before bedtime (β = 0.061), the habit of midnight snack (β = 0.055), lower body mass index (β = 0.054), NSAID users (β = 0.051), female gender (β = 0.048), lack of breakfast (β = 0.045), lack of physical exercise (β = 0.035), younger age (β = 0.033), antihyperglycemic agents non-users (β = 0.026), the habit of quick eating (β = 0.025), alcohol drinking (β = 0.025), history of gastrectomy (β = 0.024), history of cardiovascular disease (β = 0.020), and smoking (β = 0.018). Positively correlated factors for PPI users are female gender (β = 0.198), inadequate sleep (β = 0.150), lack of breakfast (β = 0.146), antihypertensive agent non-users (β = 0.134), and dinner just before bedtime (β = 0.129), whereas those for H_2_RA users are inadequate sleep (β = 0.248), habit of midnight snack (β = 0.160), anticoagulants non-users (β = 0.106), and antihypertensive agents non-users (β = 0.095).

**Conclusions:**

Among many lifestyle-related factors correlated with GERD symptoms, poor quality of sleep and irregular dietary habits are strong risk factors for high FSSG scores. At present, usual dose of PPI or H_2_RA in Japan cannot fully relieve GERD symptoms.

## Background

Gastroesophageal reflux disease (GERD) is defined as a condition of troublesome symptoms and/or complications caused from the reflux of stomach contents [1]. Despite the high morbidity rate at present, the number of GERD patients is still increasing worldwide [2]. Most GERD patients presented esophageal syndromes such as heartburn, chest pain, dysphagia, odynophagia, and so on, though it has recently become clear that not a few latent GERD subjects are suffering from extraesophageal syndromes [1]. Esophageal GERD includes two pathophysiological states: reflux esophagitis (RE, diagnosed by endoscopic observation) and non-erosive reflux disease (NERD, mainly diagnosed on the basis of the upper gastrointestinal symptoms). Most studies reported that NERD patients occupy more than half of all GERD patients [3-5].

Nowadays, it has been the worldwide consensus that the goals of GERD therapy should be based on the improvement of various symptoms and prevention of complications, such as esophageal strictures, gastrointestinal bleeding and Barrett's esophagus [6-9]. Actually, most of the guidelines for GERD emphasize relieving the bothersome symptoms rather than preventing the endoscopic esophageal injury [10-12], probably reflecting the high prevalence of endoscopy-negative cases among GERD patients [4,5]. Therefore, precise evaluation of GERD symptoms is quite important for not only grasping the patient's disorder but also assessing effectiveness of the therapy. To assess the GERD symptoms, several questionnaires have been proposed, such as QUEST [13], Manterola's Scale [14], FSSG (Frequency Scale for the Symptoms of GERD) [15], Zimmerman's Scale [16], and so forth. Whereas typical symptoms of GERD are heartburn and regurgitation [17], it is well known that GERD patients present very diverse symptoms [1]. In the present study, we chose FSSG scoring, as it can evaluate not only the acid-reflux related symptoms but also the dyspeptic symptoms [15,18].

There have been many studies which examined the relation between GERD (especially reflux esophagitis) and predictive background factors such as age [19,20], gender [19,20], body mass index (BMI) [21], obesity [21], hiatus hernia [22], and so on, but large-scale studies focusing on GERD symptoms alone have been very few. Since it is a very common disease affecting millions of people around the globe, it is quite important to clarify the causative lifestyle factors affecting various GERD symptoms. Therefore, one of our aims in this study is to analyze the correlation between GERD symptoms and background variables, especially focused on lifestyle factors.

Another aim of our study is to evaluate the efficacy of proton pump inhibitors (PPI) and histamine H_2_-receptor antagonists (H_2_RA), both of which are the most popular drugs used for GERD treatment world-wide. The FSSG scores of PPI users and H_2_RA users were analyzed in the same univariate and multivariate manner, which would illuminate actual background factors of GERD patients under medical treatment, and also could clarify the efficacy of medication upon habitual antacid users. Through the cross-sectional analysis of the large-scale healthy population, our study should shed light on the pathophysiology of GERD symptoms.

## Methods

### Study subjects

The study population was 20,773 subjects who received medical checkups at Kameda Medical Center Makuhari (Chiba-shi, Chiba, Japan) in 2010, and also approved their entry into our study. If the subject had a health checkup twice in 2010, the former data were used. Criteria for exclusion were age less than 20 years and insufficient answers to the questionnaire (22 questions under-mentioned and 10 questions from FSSG). To avoid interviewer bias, the questionnaire was self-administered for all the participants. This study was approved by the ethics committees of the University of Tokyo, and written informed consents were obtained from all study participants according to the Declaration of Helsinki.

### FSSG (Frequency Scale for the Symptoms of GERD) and questionnaire

**Figure 1:**
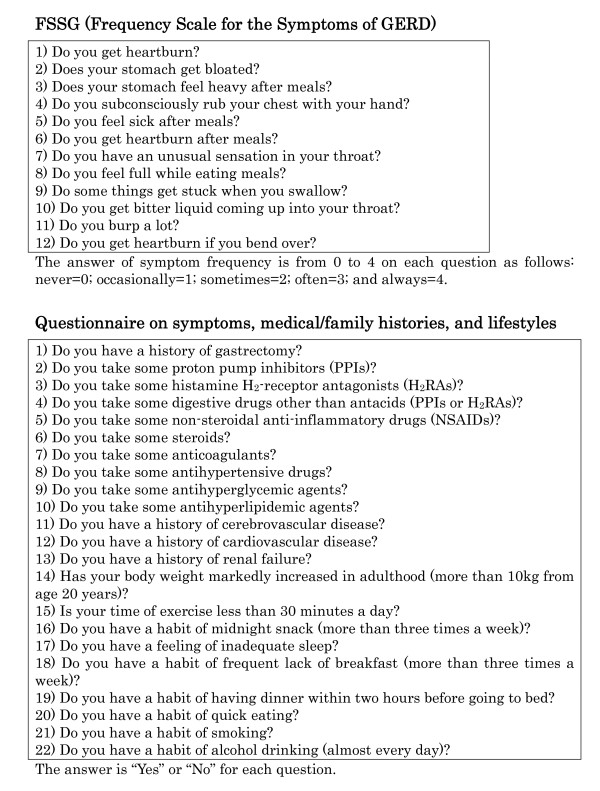
**FSSG (Frequency Scale for the Symptoms of GERD) and questionnaire used in the study**. For FSSG, the answer of symptom frequency is from 0 to 4 on each question as follows: never = 0; occasionally = 1; sometimes = 2; often = 3; and always = 4. For the questionnaire on symptoms, medical/family histories, and lifestyles, the answer is "Yes" or "No" for each question.

FSSG is a widely used questionnaire for the diagnosis of GERD [23-25], and also for evaluating the effectiveness of the treatment [15,26]. In the previous study, comparing FSSG with QUEST, it was validated that there was no difference between both questionnaires in sensitivity, specificity and accuracy for any condition [18]. The twelve questions of the FSSG cover various symptoms related to the upper gastrointestinal tract as well as psychosomatic symptoms [15] (Figure 1); a score of more than seven points suggested the presence of GERD in the respondent [15,18].

In this study, all the participants were asked to respond to the FSSG scale questionnaire, along with another detailed questionnaire consisting of 22 original questions. Additional 22 questions include enquiries about symptoms related to the upper gastrointestinal tract, medical history, family history, lifestyle factors and so on (Figure 1).

### Statistical methods

Univariate analysis was performed with the FSSG score as a response variable and 25 background factors (age, gender, BMI, and 22 answers to the questionnaires) as explanatory variables. Student's *t*-test (*P *< 0.05) or Pearson's correlation coefficient (*P *< 0.05) were used for univariate analyses. A multiple linear regression model was next applied for predictive background factors selected from the univariate analyses. A two-sided *P*-value of less than 0.05 was considered statistically significant. All statistical analyses were performed using SAS version 8.2 (SAS Institute Inc., Cary, NC, USA).

## Results

### Characteristics of study subjects

**Figure 2:**
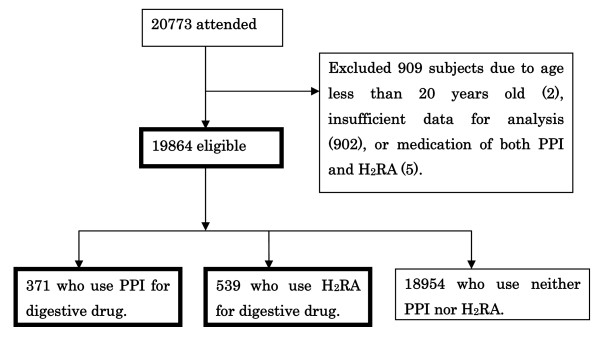
**Study recruitment flowchart**. Of the 20,773 subjects who attended this study, we excluded 909 subjects as follows: two subjects less than 20 years old, five subjects who use both PPI (proton pump inhibitor) and H_2_RA (histamine H_2_-receptor antagonists), or 902 subjects with insufficient data for analysis (incomplete answers to the questionnaires and/or FSSG, loss of critical data such as age and sex, and so on). The eligible study population of 19,864 subjects was analyzed. In addition, 371 PPI users and 539 H_2_RA users among them were also analyzed.

Of the 20,773 potential subjects for this study (Figure 2), we selected 19,864 subjects (11,493 men and 8,371 women) with a mean age of 50.2 ± 9.4 years (range 20 to 87 years). A total of 371 PPI users comprised of 239 men and 132 women with a mean age of 55.8 ± 9.9 years (range 27 to 87 years), whereas 539 H_2_RA users comprised of 332 men and 207 women with a mean age of 52.9 ± 9.8 years (range 27 to 87 years).

**Figure 3:**
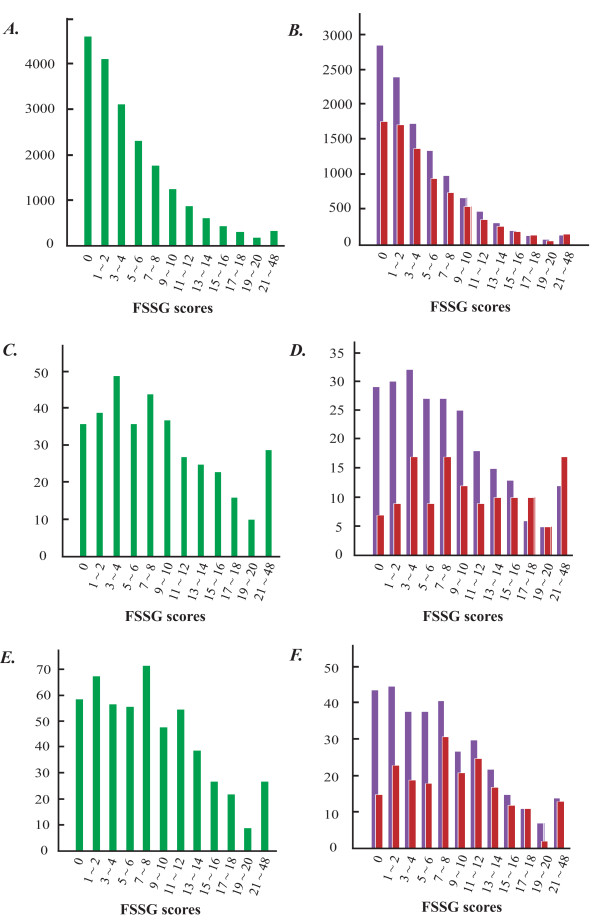
**Distribution of FSSG scores in the present study cohort**. **(A) **The FSSG score distribution of total 19,864 subjects is shown with green bars. **(B) **The FSSG score distributions of 11,943 men and 8,371 women are shown with purple bars (men) and red bars (women) respectively. **(C) **The FSSG score distribution of 371 PPI users is shown with green bars. **(D) **The FSSG score distributions of 239 male and 132 female PPI users are shown in purple and red bars respectively. **(E) **The FSSG score distribution of 539 H_2_RA users is shown with green bars. **(F) **The FSSG score distributions of 332 male and 207 female H_2_RA users are shown in purple and red bars respectively.

The distribution of FSSG scores (0 to 48) among the total subjects, PPI users, and H_2_RA users are shown in Figure 3. Average FSSG scores of subjects in the three groups are 4.8 ± 5.2 for total subjects, 9.0 ± 7.3 for PPI users, and 8.2 ± 6.6 for H_2_RA users. Both the PPI and H_2_RA users have obviously higher FSSG scores than total study participants (*P *< 0.0001).

### Positively correlated factors of FSSG scores among the total 19,864 subjects

FSSG scores of the 19,864 study subjects in the presence or absence of 25 background factors (age, BMI, gender, and answers to the 22 questions) are shown in Table 1. Based on the univariate analyses, 18 factors show positive correlation with FSSG scores: younger age, female gender, history of gastrectomy, users of digestive drugs (PPIs, H_2_RAs, and others), NSAID users, steroid users, antihyperglycemic agent non-users, history of cardiovascular disease, increased body weight in adulthood, lack of habitual physical exercise, habit of midnight snack, inadequate sleep, frequent lack of breakfast, dinner just before bedtime, the habit of quick eating, and the habit of smoking.

**Table 1:** Correlation between FSSG scores and 25 background factors of the 19,864 participants (univariate analyses).

Factors	FSSG scores of the applicable subjects to the factor	FSSG scores of the inapplicable subjects to the factor	*P-*value
Gender (female)	5.1 ± 5.4 (8,371)	4.6 ± 5.1 (11,493)	< 0.0001 *
History of gastrectomy	5.9 ± 6.0 (210)	4.8 ± 5.2 (19,654)	0.0019 *
Use of PPIs	9.0 ± 7.3 (371)	4.7 ± 5.1 (19,493)	< 0.0001 *
Use of H_2_RAs	8.2 ± 6.6 (539)	4.7 ± 5.1 (19,325)	< 0.0001 *
Use of other digestive drugs	8.2 ± 6.5 (985)	4.6 ± 5.1 (18,879)	< 0.0001 *
Use of NSAIDs	6.9 ± 5.8 (1,076)	4.7 ± 5.2 (18,788)	< 0.0001 *
Use of steroids	6.1 ± 6.1 (214)	4.8 ± 5.2 (19,650)	0.0002 *
Use of anticoagulants	4.7 ± 4.9 (572)	4.8 ± 5.2 (19,292)	0.6056
Use of antihypertensive drugs	4.6 ± 5.1 (2,581)	4.8 ± 5.2 (17,283)	0.0967
Use of antihyperglycemic agents	3.8 ± 4.3 (561)	4.8 ± 5.2 (19,303)	< 0.0001 *
Use of antihyperlipidemic agents	4.7 ± 5.2 (1,728)	4.8 ± 5.2 (18,136)	0.3572
History of cerebrovascular disease	4.8 ± 5.1 (289)	4.8 ± 5.2 (19,575)	0.9675
History of cardiovascular disease	5.6 ± 5.7 (554)	4.8 ± 5.2 (19,310)	0.0002 *
History of renal failure	4.8 ± 5.2 (86)	4.8 ± 5.2 (19,778)	0.8984
Increased body weight in adulthood	5.2 ± 5.5 (7,449)	4.5 ± 5.0 (12,415)	< 0.0001 *
Lack of habitual physical exercise	5.0 ± 5.3 (14,988)	4.1 ± 4.8 (4,876)	< 0.0001 *
Habit of midnight snack	5.9 ± 6.0 (3,180)	4.6 ± 5.0 (16,684)	< 0.0001 *
Inadequate sleep	6.0 ± 5.8 (7,988)	4.0 ± 4.6 (11,876)	< 0.0001 *
Frequent lack of breakfast	5.9 ± 6.0 (2,709)	4.6 ± 5.1 (17,155)	< 0.0001 *
Dinner just before bedtime	5.6 ± 5.8 (6,035)	4.4 ± 4.9 (13,829)	< 0.0001 *
Habit of quick eating	5.0 ± 5.3 (7,652)	4.6 ± 5.1 (12,212)	< 0.0001 *
Habit of smoking	5.0 ± 5.3 (3,981)	4.7 ± 5.2 (15,883)	0.0013 *
Habit of alcohol drinking	4.8 ± 5.2 (6,553)	4.8 ± 5.2 (13,311)	0.9618

Age	r = -0.06		< 0.0001 *
BMI	r = -0.01		0.2399

BMI, body mass index; H_2_RAs, histamine H_2_-receptor antagonists; NSAIDs, non-steroidal anti-inflammatory drugs; PPIs, proton pump inhibitors; r, regression coefficient. Except for age and BMI, the Student's *t*-test was used to evaluate the correlation between each background factor and the FSSG score (mean ± standard deviation is shown). The correlation of FSSG score with age or BMI was assessed using Pearson's correlation coefficient. The levels of significance in these univariate analyses were set at *P-*value < 0.05 (*).

We next performed the multivariate analysis, focusing on the above-mentioned univariately significant factors together with BMI and drinking. Positively correlated factors of FSSG score, in order of significance, are inadequate sleep, users of digestive drugs (PPIs, H_2_RAs, and others), increased body weight in adulthood, habitual dinner just before bedtime, habit of midnight snack, lower BMI, NSAID users, female gender, frequent lack of breakfast, lack of habitual physical exercise, younger age, antihyperglycemic agents non-users, habit of quick eating, habit of alcohol drinking, history of gastrectomy, history of cardiovascular disease, and habit of smoking (Table 2).

**Table 2:** Correlation between FSSG scores and 20 background factors of the 19,864 participants (multivariate analysis).

Factors	Regression coefficient	*P-*value	Standardized regression coefficient (β)
Inadequate sleep (†)	1.678	< 0.0001 *	0.1576
Use of other digestive drugs	2.497	< 0.0001 *	0.1038
Use of PPIs	3.749	< 0.0001 *	0.0972
Use of H_2_RAs	2.900	< 0.0001 *	0.0903
Increased body weight in adulthood	0.878	< 0.0001 *	0.0814
Dinner just before bedtime (†)	0.695	< 0.0001 *	0.0612
Habit of midnight snack (†)	0.788	< 0.0001 *	0.0554
BMI	-0.084	< 0.0001 *	-0.0535
Use of NSAIDs	1.179	< 0.0001 *	0.0512
Gender (female)	0.509	< 0.0001 *	0.0481
Frequent lack of breakfast (†)	0.683	< 0.0001 *	0.0449
Lack of habitual physical exercise (†)	0.424	< 0.0001 *	0.0350
Age	-0.018	< 0.0001 *	-0.0333
Use of antihyperglycemic agents	-0.813	0.0002 *	-0.0258
Habit of quick eating (†)	0.273	0.0002 *	0.0255
Habit of alcohol drinking (†)	0.278	0.0006 *	0.0250
History of gastrectomy	1.232	0.0004 *	0.0241
History of cardiovascular disease	0.637	0.0033 *	0.0201
Habit of smoking (†)	0.240	0.0100 *	0.0184
Use of steroids	0.303	0.3756	0.0060

BMI, body mass index; H_2_RAs, histamine H_2_-receptor antagonists; NSAIDs, non-steroidal anti-inflammatory drugs; PPIs, proton pump inhibitors. Multiple regression analysis was performed focusing on the 20 background factors, comprised of two continuous variables (age and BMI) and other 18 categorical variables. The level of significance in each factor was set at *P-*value < 0.05 (*). All the 20 background factors were sorted in order of absolute values of standardized regression coefficients. Eight lifestyle-related factors are denoted with (†).

### Positively correlated factors of FSSG scores among the PPI users

FSSG scores of the 371 PPI users in the presence or absence of 23 background factors were next analyzed (Additional file 1, Table S1). Based on the univariate analyses, 13 factors show positive correlation with FSSG scores: female gender, users of other digestive drugs, anticoagulants non-users, antihypertensive agents non-users, antihyperglycemic agents users, antihyperlipidemic agents non-users, history of cerebrovascular disease, lack of habitual physical exercise, habit of midnight snack, inadequate sleep, frequent lack of breakfast and dinner just before bedtime.

This result was followed by the multivariate analysis, focusing on the abovementioned significant 13 factors and four other essential factors (BMI, NSAID users, drinking and smoking). Among the PPI users, positively correlated factors of FSSG score in order of significance are female gender, inadequate sleep, frequent lack of breakfast, antihypertensive agent non-users, and dinner just before bedtime (Table 3).

**Table 3:** Multivariately analyzed correlation between FSSG scores and 17 background factors among 371 PPI users.

Factors	Regression coefficient	*P-*value	Standardized regression coefficient (β)
Gender (female)	3.028	0.0003 *	0.1978
Inadequate sleep	2.211	0.0027 *	0.1500
Frequent lack of breakfast	3.414	0.0034 *	0.1460
Use of antihypertensive drugs	-2.043	0.0154 *	-0.1343
Dinner just before bedtime	2.130	0.0138 *	0.1293
History of cerebrovascular disease	-3.099	0.0863	-0.0833
Age	-0.059	0.1521	-0.0788
Use of antihyperlipidemic agents	-1.135	0.1683	-0.0707
Use of antihyperglycemic agents	-1.598	0.2357	-0.0576
Habit of midnight snack	0.658	0.5204	0.0324
Use of NSAIDs	-0.750	0.5268	-0.0310
Use of anticoagulants	-0.572	0.6108	-0.0279
Lack of habitual physical exercise	0.327	0.6758	0.0202
Habit of smoking	-0.333	0.7385	-0.0169
Habit of alcohol drinking	0.262	0.7477	0.0167
Use of other digestive drugs	-0.255	0.7515	-0.0156
BMI	0.013	0.9032	0.0063

BMI, body mass index; NSAIDs, non-steroidal anti-inflammatory drugs; PPIs, proton pump inhibitors. Multiple regression analysis was performed focusing on the 17 background factors, comprised of two continuous variables (age and BMI) and other 15 categorical variables. The level of significance in each factor was set at *P-*value < 0.05 (*). All the 17 background factors were sorted in order of absolute values of standardized regression coefficients (β).

### Positively correlated factors of FSSG scores among the 539 H_2_RA users

FSSG scores of 539 H_2_RA users in the presence or absence of 23 background factors were further analyzed (Additional file 1, Table S2). Based on the univariate analyses, positively correlated factors of the FSSG score are younger age, female gender, anticoagulants non-users, antihypertensive agent non-users, history of cerebrovascular disease, history of cardiovascular disease, habit of midnight snack, inadequate sleep, frequent lack of breakfast and dinner just before bedtime.

This result was followed by the multivariate analysis, focusing on the abovementioned significant 10 factors and 4 other essential factors (BMI, NSAID users, alcohol drinking, and smoking). Among the H_2_RA users, positively correlated factors of FSSG score in order of significance are inadequate sleep, habit of midnight snack, anticoagulant non-users and antihypertensive agent non-users (Table 4).

**Table 4:** Multivariately analyzed correlation between FSSG scores and 14 background factors among 539 H_2_RA users.

Factors	Regression coefficient	*P-*value	Standardized regression coefficient (β)
Inadequate sleep	3.280	< 0.0001 *	0.2477
Habit of midnight snack	2.830	0.0001 *	0.1595
Use of anticoagulants	-2.203	0.0493 *	-0.1056
Use of antihypertensive drugs	-1.372	0.0429 *	-0.0945
Gender (female)	1.100	0.0887	0.0816
Use of NSAIDs	-1.441	0.0805	-0.0743
Age	-0.050	0.1020	-0.0742
Habit of smoking	0.666	0.3184	0.0425
Dinner just before bedtime	0.505	0.4290	0.0359
Frequent lack of breakfast	0.483	0.5296	0.0268
BMI	0.031	0.6862	0.0173
History of cardiovascular disease	0.390	0.7445	0.0161
Habit of alcohol drinking	0.167	0.7826	0.0123
History of cerebrovascular disease	-0.030	0.9821	-0.0010

BMI, body mass index; H_2_RAs, histamine H_2_-receptor antagonists; NSAIDs, non-steroidal anti-inflammatory drugs. Multiple regression analysis was performed focusing on the 14 background factors, comprised of two continuous variables (age and BMI) and other 12 categorical variables. The level of significance in each factor was set at *P-*value < 0.05 (*). All the 14 background factors were sorted in order of absolute values of standardized regression coefficients (β).

## Discussion

### Many lifestyle-related factors show strong correlation with GERD symptoms

Of the 19 significant factors, 8 are obvious lifestyle factors (Table 2). "Increased body weight in adulthood" and "BMI" are also strongly related to the subject's lifestyle. Therefore, except for use of digestive drugs (PPIs, H_2_RAs, and others), the top five correlated factors with GERD symptoms are lifestyle-related (Table 2). Alcohol consumption and habitual smoking have been reported to be putative risk factors for GERD [20], though it has still been controversial for their association with GERD "symptoms". Our present cross-sectional study demonstrated significant correlation of alcohol and smoking with GERD symptoms, but their influences upon FSSG scores were not so strong: the other eight lifestyle-related factors had much stronger influence on GERD symptoms (Table 2).

For BMI and obesity, our result was interesting; increased body weight in adulthood is a strong risk factor of GERD, whereas the BMI is negatively correlated with FSSG scores (Table 2). Unlike most previous studies [27-29], showing the positive correlation of obesity or overweight with erosive reflux esophagitis and Barrett's esophagitis, mere symptoms of GERD were evaluated in our study. Therefore, subjects with higher FSSG scores should comprise not only the reflux esophagitis (RE) patients, but also much higher numbers of patients suffering from NERD. The above-mentioned intriguing results for BMI and body weight gain may reflect the fact that subjects with GERD symptoms include two pathophysiologically entirely different disorders: reflux esophagitis (RE) and non-erosive reflux disease (NERD).

### Poor quality of sleep and irregular dietary habits are the most significant risk factors for GERD symptoms

From our results, it is suggested that good quality of sleep, orderly eating habits and body weight control are important for avoiding GERD symptoms. In particular, inadequate sleep is a very strong background factor, not only in the total population (Table 2) but also among the antacid users (Tables 3 and 4). Nocturnal GERD is thought to be caused by such things as a decrease in esophageal peristalsis, diminished salivary production during sleep, decline of the upper esophageal sphincter basal pressure, reduced conscious-dependent behavior during sleep, distended stomach due to intragastric food, frequent lower esophageal sphincter relaxation in the supine position, and so on [30,31]. It is well-known that nighttime GERD symptoms are the crucial cause of sleep disorders [32,33], but recent studies also suggested that a link between sleep problems and GERD might be bidirectional, for example, due to the influence of sleep stages on esophago-upper esophageal sphincter contractile reflex [34] or due to reinforcing perception of intra-esophageal acid [35]. Therefore, improving quality of sleep might be essential for relieving GERD symptoms.

Our results also indicated that dietary habits have significant correlation with FSSG scores (Table 2). Although there have been very few reports showing the effect of dietary habits upon pathogenesis of GERD [31], our results showed an obvious correlation between GERD symptoms and dietary habits. Accordingly, such dietary habits as the following should be avoided: 1) having dinner a few hours before going to bed, 2) the habit of eating a midnight snack, 3) frequently going without breakfast, and 4) the habit of quick eating. It should be noteworthy that these four diet-related factors present more significant effects than alcohol or smoking on GERD symptoms (Table 2).

### Medication of PPIs or H_2_RAs is not enough to relieve the GERD symptoms

Among 19 correlated background factors, medication with digestive drugs (PPIs, H_2_RAs and others) shows a high correlation with FSSG scores (Table 2). Based on a meta-analysis of many trials compared with placebo [19], both PPIs and H_2_RAs have been proved to be effective in the treatment of reflux esophagitis (RE). These two antacids are the common drugs for GERD treatment, and it has been proved that PPIs are better than H_2_RAs in the treatment of RE [19]. As for NERD, it has also been reported that PPIs are better than H_2_RAs and placebo, although the effect of antacid medication upon NERD patients is smaller than that upon RE patients [36]. Based on these many previous studies, almost all guidelines worldwide recommend the use of antacids, especially PPIs [11,12,37].

Our results clearly demonstrate that many patients with digestive drug medication (PPIs, H_2_RAs, and others) are suffering from GERD symptoms reflected as high FSSG scores. In other words, it is indicated that present-day digestive medicine could not fully relieve the GERD symptoms. For both the PPI users and H_2_RA users (Tables 3 and 4), inadequate sleep and some dietary habits show significant correlation with GERD symptoms, which suggests that improving the quality of sleep and ordered dietary habits should be recommended to them. Actually, in Japan today, stronger antacids, such as higher doses of PPIs, are eagerly expected by many gastroenterologists in clinical situations and/or daily bedside visits. Our results suggest that development of more effective digestive drugs or improvement of the usage of present-day agents is necessary.

### Characteristic background factors correlated to FSSG scores among PPI users and H_2_RA users

It should be noted that there are some distinctive characteristics for background factors of PPI users and H_2_RA users (Tables 3 and 4). One is the marked difference in the correlation of gender; female gender is the strongest background factor of the 371 PPI users, whereas it shows no significant correlation among the 539 H_2_RA users. It may reflect that the rates of subjects with the most severe GERD symptoms (FSSG scores of 21 to 48) were high in women (1.89%) compared with men (1.31%). At present, however, the precise mechanism of the difference between PPI users and H_2_RA users is not elucidated.

Another characteristic is the positive correlation of the use of antihypertensive drugs for GERD symptoms in both PPI users and H_2_RA users (Tables 3 and 4). Though there have been very few studies showing the association of GERD with hypertension or antihypertensive drug use, a cross-sectional study from Japan reported positive correlation between hypertension and reflux esophagitis [38]. They speculated the effect of decreasing the lower esophageal sphincter pressure by calcium antagonists, the most frequently used antihypertensive drugs in Japan [38,39]. In our results, however, the significant correlation of antihypertensive drug use was detected in PPI users and H_2_RA users only, and not detected in the total 19,864 subjects. We have no speculation for this correlation at present; statistical analysis is needed in the future, together with precise data of the kind of antihypertensive drugs and measured blood pressures of the subjects.

### Study limitation

One of limitations of our study was that the study subjects may tend to be comprised of rather wealthy people, as the fee for participating in the medical checkup program was not too expensive but also not too cheap. Therefore, our study result could not completely reflect the population-based data of Japanese or East Asian people. Another limitation of our study was the lack of information on dose and type of PPIs and H_2_RAs. More detailed information of orally taken antacids might more precisely show their influence on FSSG scores.

## Conclusion

A large-scale study in Japan revealed that many lifestyle-related factors have correlation with high scores of FSSG (Frequency Scale for the Symptoms of GERD). Poor quality of sleep and irregular dietary habits are the strongest risk factors among them. The present-day usual dose of PPI or H_2_RA cannot fully relieve GERD symptoms.

## Abbreviations

BMI: body mass index; FSSG: Frequency Scale for the Symptoms of GERD; GERD: gastroesophageal reflux disease; H_2_RA: histamine H_2 _receptor antagonist; NERD: non-erosive reflux disease; NSAID: non-steroidal anti-inflammatory drugs; PPI: proton pump inhibitor; RE: reflux esophagitis.

## Competing interests

The authors declare that they have no competing interests.

## Authors' contributions

NY contributed to the study concept and design, acquisition of data, analysis and interpretation of data, statistical analysis, and drafting of the manuscript. SM participated in critical revision of the manuscript for important intellectual content, analysis and interpretation of data. IA critically revised the manuscript for important intellectual content. RM contributed to acquisition of data, while TS participated in support of statistical analysis. MK, CT, KN, SO, SK and CMacquired data, YT analyzed and interpreted data. MF contributed to the critical revision of the manuscript for important intellectual content and to administrative support. TM and KK participated in study concept and design and study supervision. We confirm that all authors checked and approved the final version of the manuscript.

## Pre-publication history

The pre-publication history for this paper can be accessed here:

http://www.biomedcentral.com/1741-7015/10/45/prepub

## Supplementary Material

Additional files 1 **Two tables showing univariately analyzed correlation between FSSG scores and 23 background factors among 371 PPI users (Table S1) or 539 H_2_RAs users (Table S2)**. Click here for file

## References

[B1] Vakil N van Zanten SV Kahrilas P Dent J Jones R The Montreal definition and classification of gastroesophageal reflux disease: a global evidence-based consensus Am J Gastroenterol 2006 101 1900 1920 quiz 1943 16928254 10.1111/j.1572-0241.2006.00630.x

[B2] el-Serag HB Sonnenberg A Opposing time trends of peptic ulcer and reflux disease Gut 1998 43 327 333 9863476 10.1136/gut.43.3.327 PMC1727258

[B3] Lind T Havelund T Carlsson R Anker-Hansen O Glise H Hernqvist H Junghard O Lauritsen K Lundell L Pedersen SA Stubberod A Heartburn without oesophagitis: efficacy of omeprazole therapy and features determining therapeutic response Scand J Gastroenterol 1997 32 974 979 9361168 10.3109/00365529709011212

[B4] Fass R Fennerty MB Vakil N Nonerosive reflux disease--current concepts and dilemmas Am J Gastroenterol 2001 96 303 314 11232668 10.1111/j.1572-0241.2001.03511.x

[B5] El-Serag HB Epidemiology of non-erosive reflux disease Digestion 2008 78 Suppl 1 6 10 18832834 10.1159/000151249

[B6] Bytzer P Goals of therapy and guidelines for treatment success in symptomatic gastroesophageal reflux disease patients Am J Gastroenterol 2003 98 S31 39 12644029 10.1016/s0002-9270(03)00013-3

[B7] Pisegna J Holtmann G Howden CW Katelaris PH Sharma P Spechler S Triadafilopoulos G Tytgat G Review article: oesophageal complications and consequences of persistent gastro-oesophageal reflux disease Aliment Pharmacol Ther 2004 20 Suppl 9 47 56 15527464 10.1111/j.1365-2036.2004.02240.x PMC6736593

[B8] Meineche-Schmidt V Juhl HH Ostergaard JE Luckow A Hvenegaard A Costs and efficacy of three different esomeprazole treatment strategies for long-term management of gastro-oesophageal reflux symptoms in primary care Aliment Pharmacol Ther 2004 19 907 915 15080852 10.1111/j.1365-2036.2004.01916.x

[B9] Pace F Negrini C Wiklund I Rossi C Savarino V Quality of life in acute and maintenance treatment of non-erosive and mild erosive gastro-oesophageal reflux disease Aliment Pharmacol Ther 2005 22 349 356 16098002 10.1111/j.1365-2036.2005.02558.x

[B10] Fujiwara Y Takahashi S Arakawa T Sollano JD Zhu Q Kachintorn U Rani AA Hahm KB Joh T Kinoshita Y Matsumoto T Naito Y Takeuchi K Furuta K Terano A A 2008 questionnaire-based survey of gastroesophageal reflux disease and related diseases by physicians in East Asian countries Digestion 2009 80 119 128 19641321 10.1159/000226088

[B11] DeVault KR Castell DO Updated guidelines for the diagnosis and treatment of gastroesophageal reflux disease Am J Gastroenterol 2005 100 190 200 15654800 10.1111/j.1572-0241.2005.41217.x

[B12] Katelaris P Holloway R Talley N Gotley D Williams S Dent J Gastro-oesophageal reflux disease in adults: Guidelines for clinicians J Gastroenterol Hepatol 2002 17 825 833 12164956 10.1046/j.1440-1746.2002.02839.x

[B13] Carlsson R Dent J Bolling-Sternevald E Johnsson F Junghard O Lauritsen K Riley S Lundell L The usefulness of a structured questionnaire in the assessment of symptomatic gastroesophageal reflux disease Scand J Gastroenterol 1998 33 1023 1029 9829354 10.1080/003655298750026697

[B14] Manterola C Munoz S Grande L Bustos L Initial validation of a questionnaire for detecting gastroesophageal reflux disease in epidemiological settings J Clin Epidemiol 2002 55 1041 1045 12464381 10.1016/s0895-4356(02)00454-7

[B15] Kusano M Shimoyama Y Sugimoto S Kawamura O Maeda M Minashi K Kuribayashi S Higuchi T Zai H Ino K Horikoshi T Sugiyama T Toki M Ohwada T Mori M Development and evaluation of FSSG: frequency scale for the symptoms of GERD J Gastroenterol 2004 39 888 891 15565409 10.1007/s00535-004-1417-7

[B16] Zimmerman J Validation of a brief inventory for diagnosis and monitoring of symptomatic gastro-oesophageal reflux Scand J Gastroenterol 2004 39 212 216 15074388 10.1080/00365520310005333

[B17] Klauser AG Schindlbeck NE Muller-Lissner SA Symptoms in gastro-oesophageal reflux disease Lancet 1990 335 205 208 1967675 10.1016/0140-6736(90)90287-f

[B18] Danjo A Yamaguchi K Fujimoto K Saitoh T Inamori M Ando T Shimatani T Adachi K Kinjo F Kuribayashi S Mitsufuji S Fujiwara Y Koyama S Akiyama J Takagi A Manabe N Miwa H Shimoyama Y Kusano M Comparison of endoscopic findings with symptom assessment systems (FSSG and QUEST) for gastroesophageal reflux disease in Japanese centres J Gastroenterol Hepatol 2009 24 633 638 19220681 10.1111/j.1440-1746.2008.05747.x

[B19] Moayyedi P Talley NJ Gastro-oesophageal reflux disease Lancet 2006 367 2086 2100 16798392 10.1016/S0140-6736(06)68932-0

[B20] Dent J El-Serag HB Wallander MA Johansson S Epidemiology of gastro-oesophageal reflux disease: a systematic review Gut 2005 54 710 717 15831922 10.1136/gut.2004.051821 PMC1774487

[B21] Lagergren J Influence of obesity on the risk of esophageal disorders Nat Rev Gastroenterol Hepatol 2011 8 340 347 21643038 10.1038/nrgastro.2011.73

[B22] Zagari RM Fuccio L Wallander MA Johansson S Fiocca R Casanova S Farahmand BY Winchester CC Roda E Bazzoli F Gastro-oesophageal reflux symptoms, oesophagitis and Barrett's oesophagus in the general population: the Loiano-Monghidoro study Gut 2008 57 1354 1359 18424568 10.1136/gut.2007.145177

[B23] Yasaka S Murakami K Abe T Anan J Mizukami K Tanahashi J Okimoto T Kodama M Kudo Y Kawasaki H Fujioka T Evaluation of esophageal function in patients with gastroesophageal reflux disease using transnasal endoscopy J Gastroenterol Hepatol 2009 24 1677 1682 19788608 10.1111/j.1440-1746.2009.05973.x

[B24] Miyamoto M Haruma K Takeuchi K Kuwabara M Frequency scale for symptoms of gastroesophageal reflux disease predicts the need for addition of prokinetics to proton pump inhibitor therapy J Gastroenterol Hepatol 2008 23 746 751 18028348 10.1111/j.1440-1746.2007.05218.x

[B25] Furuta T Shimatani T Sugimoto M Ishihara S Fujiwara Y Kusano M Koike T Hongo M Chiba T Kinoshita Y Investigation of pretreatment prediction of proton pump inhibitor (PPI)-resistant patients with gastroesophageal reflux disease and the dose escalation challenge of PPIs-TORNADO study: a multicenter prospective study by the Acid-Related Symptom Research Group in Japan J Gastroenterol 2011 46 1273 1283 21861141 10.1007/s00535-011-0446-2

[B26] Sakamoto Y Inamori M Iwasaki T Lida H Endo H Hosono K Ikeda T Fujita K Yoneda M Takahashi H Koide T Tokoro C Goto A Abe Y Kirikoshi H Kobayashi N Kubota K Saito S Nakajima A Relationship between upper gastrointestinal symptoms and diet therapy: examination using frequency scale for the symptoms of gastroesophageal reflux disease Hepatogastroenterology 2010 57 1635 1638 21443134

[B27] Hampel H Abraham NS El-Serag HB Meta-analysis: obesity and the risk for gastroesophageal reflux disease and its complications Ann Intern Med 2005 143 199 211 16061918 10.7326/0003-4819-143-3-200508020-00006

[B28] Friedenberg FK Xanthopoulos M Foster GD Richter JE The association between gastroesophageal reflux disease and obesity Am J Gastroenterol 2008 103 2111 2122 18796104 10.1111/j.1572-0241.2008.01946.x

[B29] Corley DA Kubo A Body mass index and gastroesophageal reflux disease: a systematic review and meta-analysis Am J Gastroenterol 2006 101 2619 2628 16952280 10.1111/j.1572-0241.2006.00849.x

[B30] Fass R Effect of gastroesophageal reflux disease on sleep J Gastroenterol Hepatol 2010 25 Suppl 1 S41 44 20586864 10.1111/j.1440-1746.2009.06210.x

[B31] Fujiwara Y Machida A Watanabe Y Shiba M Tominaga K Watanabe T Oshitani N Higuchi K Arakawa T Association between dinner-to-bed time and gastro-esophageal reflux disease Am J Gastroenterol 2005 100 2633 2636 16393212 10.1111/j.1572-0241.2005.00354.x

[B32] Jansson C Nordenstedt H Wallander MA Johansson S Johnsen R Hveem K Lagergren J A population-based study showing an association between gastroesophageal reflux disease and sleep problems Clin Gastroenterol Hepatol 2009 7 960 965 19286481 10.1016/j.cgh.2009.03.007

[B33] Mody R Bolge SC Kannan H Fass R Effects of gastroesophageal reflux disease on sleep and outcomes Clin Gastroenterol Hepatol 2009 7 953 959 19375520 10.1016/j.cgh.2009.04.005

[B34] Bajaj JS Bajaj S Dua KS Jaradeh S Rittmann T Hofmann C Shaker R Influence of sleep stages on esophago-upper esophageal sphincter contractile reflex and secondary esophageal peristalsis Gastroenterology 2006 130 17 25 16401464 10.1053/j.gastro.2005.10.003

[B35] Schey R Dickman R Parthasarathy S Quan SF Wendel C Merchant J Powers J Han B van Handel D Fass R Sleep deprivation is hyperalgesic in patients with gastroesophageal reflux disease Gastroenterology 2007 133 1787 1795 18054551 10.1053/j.gastro.2007.09.039

[B36] van Pinxteren B Numans ME Bonis PA Lau J Short-term treatment with proton pump inhibitors, H2-receptor antagonists and prokinetics for gastro-oesophageal reflux disease-like symptoms and endoscopy negative reflux disease Cochrane Database Syst Rev 2004 CD002095 15495027 10.1002/14651858.CD002095.pub2

[B37] Japanese Society of Gastroenterology Gastroesophageal reflux Nihon Shokakibyo Gakkai Zasshi 2009 Suppl I 1 119 21434450

[B38] Moki F Kusano M Mizuide M Shimoyama Y Kawamura O Takagi H Imai T Mori M Association between reflux oesophagitis and features of the metabolic syndrome in Japan Aliment Pharmacol Ther 2007 26 1069 1075 17877514 10.1111/j.1365-2036.2007.03454.x

[B39] Hongo M Traube M McAllister RG Jr McCallum RW Effects of nifedipine on esophageal motor function in humans: correlation with plasma nifedipine concentration Gastroenterology 1984 86 8 12 6689676

