# Supplementary material for: Lifestyle factors affecting gastroesophageal reflux disease symptoms: a cross-sectional study of healthy 19864 adults using FSSG scores
Source: BMC Med. 2012 May 3;10:45. doi: 10.1186/1741-7015-10-45 (PMC3353848; doi:10.1186/1741-7015-10-45)
Supplement: Additional files 1 — Two tables showing univariately analyzed correlation between FSSG scores and 23 background factors among 371 PPI users (Table S1) or 539 H2RAs users (Table S2). [file 1741-7015-10-45-S1.DOC]

Table S1. Univariately analyzed correlation between FSSG scores and 23 background factors among 371 PPI users.

| **Factors** | **FSSG scores of the applicable subjects to the factor** | **FSSG scores of the inapplicable subjects to the factor** | ***p* value** |
| --- | --- | --- | --- |
| Gender (female) | 11.2 ± 8.0 (132) | 7.8 ± 6.7 (239) | <0.0001 * |
| History of gastrectomy | 9.5 ± 6.9 (8) | 9.0 ± 7.4 (363) | 0.8442 |
| Use of other digestive drugs | 10.2 ± 8.1 (104) | 8.5 ± 7.0 (267) | 0.0479 * |
| Use of NSAIDs | 10.3 ± 8.0 (38) | 8.8 ± 7.3 (333) | 0.2516 |
| Use of steroids | 7.7 ± 10.1 (21) | 9.1 ± 7.2 (350) | 0.4112 |
| Use of anticoagulants | 5.3 ± 6.0 (56) | 9.7 ± 7.4 (315) | <0.0001 * |
| Use of antihypertensive drugs | 6.4 ± 5.8 (136) | 10.5 ± 7.7 (235) | <0.0001 * |
| Use of antihyperglycemic agents | 5.8 ± 4.8 (28) | 4.8 ± 5.2 (343) | 0.0172 * |
| Use of antihyperlipidemic agents | 7.0 ± 6.4 (110) | 9.8 ± 7.5 (261) | 0.0063 * |
| History of cerebrovascular disease | 5.0 ± 6.5 (15) | 9.2 ± 7.3 (356) | 0.0312 * |
| History of cardiovascular disease | 7.1 ± 6.9 (43) | 9.2 ± 7.4 (328) | 0.0743 |
| History of renal failure | 9.4 ± 8.9 (5) | 9.0 ± 7.3 (366) | 0.9012 |
| Increased body weight in adulthood | 9.0 ± 7.9 (199) | 9.0 ± 6.7 (172) | 0.9895 |
| Lack of habitual physical exercise | 9.5 ± 7.5 (263) | 7.8 ± 6.8 (108) | 0.0421 * |
| Habit of midnight snack | 11.0 ± 8.4 (57) | 8.6 ± 7.1 (314) | 0.0260 * |
| Inadequate sleep | 11.2 ± 8.1 (165) | 7.2 ± 6.1 (206) | <0.0001 * |
| Frequent lack of breakfast | 13.7 ± 9.8 (41) | 8.4 ± 6.8 (330) | <0.0001 * |
| Dinner just before bedtime | 11.4 ± 8.4 (101) | 8.1 ± 6.7 (270) | <0.0001 * |
| Habit of quick eating | 9.2 ± 6.9 (142) | 8.8 ± 7.6 (229) | 0.6136 |
| Habit of smoking | 8.7 ± 5.8 (61) | 9.1 ± 7.6 (310) | 0.7221 |
| Habit of alcohol drinking | 8.4 ± 6.4 (120) | 9.3 ± 7.8 (251) | 0.2614 |
| Age | r = -0.26 | | <0.0001 * |
| BMI | r = -0.04 | | 0.4022 |

PPIs, proton pump inhibitors; NSAIDs, non-steroidal anti-inflammatory drugs; BMI, body mass index; r, regression coefficient. Except for age and BMI, the Student's T-test was used to evaluate the correlation between each background factor and the FSSG score (mean ± standard deviation is shown). The correlation of FSSG score with age or BMI was assessed using Pearson's correlation coefficient. The levels of significance in these univariate analyses were set at *p* value <0.05 (*).

Table S2. Univariately analyzed correlation between FSSG scores and 23 background factors among 539 H_2_RAs users.

| **Factors** | **FSSG scores of the applicable subjects to the factor** | **FSSG scores of the inapplicable subjects to the factor** | ***p* value** |
| --- | --- | --- | --- |
| Gender (female) | 9.2 ± 6.7 (207) | 7.6 ± 6.4 (332) | 0.0039 * |
| History of gastrectomy | 7.3 ± 6.5 (12) | 8.2 ± 6.6 (527) | 0.6405 |
| Use of other digestive drugs | 9.0 ± 6.3 (141) | 7.9 ± 6.6 (398) | 0.1056 |
| Use of NSAIDs | 8.1 ± 6.3 (71) | 8.2 ± 6.6 (468) | 0.9092 |
| Use of steroids | 6.6 ± 7.2 (23) | 8.3 ± 6.5 (516) | 0.2323 |
| Use of anticoagulants | 5.1 ± 5.2 (60) | 8.6 ± 6.6 (479) | <0.0001 * |
| Use of antihypertensive drugs | 6.3 ± 5.3 (154) | 9.0 ± 6.9 (385) | <0.0001 * |
| Use of antihyperglycemic agents | 6.7 ± 5.6 (30) | 8.3 ± 6.6 (509) | 0.2053 |
| Use of antihyperlipidemic agents | 7.1 ± 6.3 (103) | 8.5 ± 6.6 (436) | 0.0537 |
| History of cerebrovascular disease | 5.4 ± 4.4 (25) | 8.3 ± 6.6 (514) | 0.0306 * |
| History of cardiovascular disease | 6.0 ± 7.0 (43) | 8.4 ± 6.5 (496) | 0.0199 * |
| History of renal failure | 1.0 ± 1.4 (2) | 8.2 ± 6.6 (537) | 0.1198 |
| Increased body weight in adulthood | 8.3 ± 6.5 (234) | 8.1 ± 6.6 (305) | 0.6728 |
| Lack of habitual physical exercise | 8.6 ± 6.8 (385) | 7.4 ± 6.0 (154) | 0.0565 |
| Habit of midnight snack | 10.8 ± 7.5 (88) | 7.7 ± 6.2 (451) | <0.0001 * |
| Inadequate sleep | 10.5 ± 7.2 (233) | 6.5 ± 5.4 (306) | <0.0001 * |
| Frequent lack of breakfast | 9.9 ± 7.3 (85) | 7.9 ± 6.4 (454) | 0.0109 * |
| Dinner just before bedtime | 9.4 ± 6.8 (173) | 7.7 ± 6.4 (366) | 0.0039 * |
| Habit of quick eating | 7.9 ± 6.5 (201) | 8.4 ± 6.6 (338) | 0.4632 |
| Habit of smoking | 8.8 ± 6.4 (122) | 8.0 ± 6.6 (417) | 0.2768 |
| Habit of alcohol drinking | 7.9 ± 6.4 (198) | 8.4 ± 6.6 (341) | 0.4343 |
| Age | r = -0.18 | | <0.0001 * |
| BMI | r = -0.05 | | 0.2155 |

H_2_RAs, histamine H_2_-receptor antagonists; NSAIDs, non-steroidal anti-inflammatory drugs; BMI, body mass index; r, regression coefficient. Except for age and BMI, the Student's T-test was used to evaluate the correlation between each background factor and the FSSG score (mean ± standard deviation is shown). The correlation of FSSG score with age or BMI was assessed using Pearson's correlation coefficient. The levels of significance in these univariate analyses were set at *p* value <0.05 (*).
